# QSYQ Attenuates Oxidative Stress and Apoptosis Induced Heart Remodeling Rats through Different Subtypes of NADPH-Oxidase

**DOI:** 10.1155/2013/824960

**Published:** 2013-06-04

**Authors:** Yong Wang, Chun Li, Yuli Ouyang, Tianjiao Shi, Xiaomin Yang, Junda Yu, Qi Qiu, Jing Han, Yan Wu, Binghua Tang, Wei Wang

**Affiliations:** ^1^Beijing University of Chinese Medicine, Bei San Huan Dong Lu 11, Chao Yang District, 100029 Beijing, China; ^2^Capital Medical University Beijing Anzhen Hospital, Beijing, China

## Abstract

We aim to investigate the therapeutic effects of QSYQ, a drug of heart failure (HF) in clinical practice in China, on a rat heart failure (HF) model. 3 groups were divided: HF model group (LAD ligation), QSYQ group (LAD ligation and treated with QSYQ), and sham-operated group. After 4 weeks, rats were sacrificed for cardiac injury measurements. Rats with HF showed obvious histological changes including necrosis and inflammation foci, elevated ventricular remodeling markers levels(matrix metalloproteinases-2, MMP-2), deregulated ejection fraction (EF) value, increased formation of oxidative stress (Malondialdehyde, MDA), and up-regulated levels of apoptotic cells (caspase-3, p53 and tunnel) in myocardial tissue. Treatment of QSYQ improved cardiac remodeling through counter-acting those events. The improvement of QSYQ was accompanied with a restoration of NADPH oxidase 4 (NOX4) and NADPH oxidase 2 (NOX2) pathways in different patterns. Administration of QSYQ could attenuate LAD-induced HF, and AngII-NOX2-ROS-MMPs pathway seemed to be the critical potential targets for QSYQ to reduce the remodeling. Moreover, NOX4 was another key targets to inhibit the p53 and Caspase3, thus to reduce the hypertrophy and apoptosis, and eventually provide a synergetic cardiac protective effect.

## 1. Introduction

Heart failure (HF) after myocardial infarction (MI) is a common clinical syndrome with high morbidity and mortality [1]. It has been suggested that HF is closely correlated with cardiac remodeling [2]. A variety of known factors are involved in the development and progression of HF [3]; during these factors, reactive oxygen species (ROS) and apoptosis were considered as the most critical pathological changes [4]. 

More and more pieces of evidence have indicated that the activated oxidative stress contributes significantly to the deterioration of cardiovascular function and eventually leads to myocardial remodeling [5]. Experimental and clinical heart failure trials documented an increased production of ROS, like superoxide, malondialdehyde, and hydroxyl radicals. Different sources of increased ROS production were found in the failing heart, including NAD(P)H oxidase (NOX) [6]. Two NOX isoforms, NOX2 and NOX4, are deeply investigated. Studies using NOX2 activity deficiency mice indicated that NOX2 activation contributes to angiotensin-II-induced cardiomyocyte hypertrophy [7]. In contrast, NOX4, which is constitutively active at a low level, can be induced by pressure overload and mediates cardiac hypertrophy mainly by apoptosis [8]. The development to target individual NOX isoforms may be important for the achievement of therapeutic efficacy in heart failure [9].

Apoptosis is another critical pathologic change in HF. In particular, apoptotic cell death in infarcted tissue is reported to play an important role in progression of cardiac remodeling in HF [10]. Hayakawa et al. have reported that inhibition of cell apoptosis significantly improved left ventricular remodeling and heart failure in the chronic stage [11]. Especially, p53-caspase-3 mediated apoptosis is considered to be the critical pathway during the HF [12]. Therefore not in vivo detection of apoptosis only may prove clinically useful information in the diagnosis and prognosis but also indicates that p53 pathway may be the potential target for the drugs [13]. Furthermore, apoptosis can be triggered by NAD(P)H oxidase-ROS pathway, which makes HF more complicated.

QiShenYiQi (QSYQ), a formula long been used for the routine treatment of coronary heart disease (CHD) or chronic heart failure (CHF) in China, has benefit efficacy in clinical [14]. It consists of six Chinese herbs (Radix Astragali Mongolici, *Salvia miltiorrhiza* bunge, Flos Lonicerae, *Scrophularia*, Radix Aconiti Lateralis Preparata, and Radix Glycyrrhizae), and is widely produced in China in accordance with the China Pharmacopoeia standard of quality control [13]. Our previous study found that QSYQ ameliorated myocardial hypertrophy and remodeling by inhibiting the expression of AngII in LAD rats [15]. However, little is known about the exact targets of QSYQ acting on myocardial remodeling. The purpose of the present study is to investigate whether QSYQ can act on HF in improving left ventricular remodeling associated with oxidative stress and apoptosis. 

## 2. Materials and Methods

### 2.1. Animals and Grouping

Studies were performed in accordance with the China Physiological Society's “Guiding Principles in the Care and Use of Animals” and with approval of the Animal Care Committee of Beijing Medical Center. A total of 60 male SD rats (weighted 240 ± 10 g) in SPF grade were selected (purchased from Beijing Vital River Laboratory Animal Technology Co. Ltd.) into the study. Rats were housed in a standard animal room with food and water ad libitum under controlled conditions of humidity and temperature (25 ± 1.2°C), under a 12 h light : 12 h dark lighting schedule.

### 2.2. HF Model Preparation

HF model was established as before [14]. Briefly, pentobarbital-anesthetized rats were fixed on the operating table. The thoracic cavity was opened to expose the heart, and the left coronary arteries (LADs) were ligated. In the sham group, the sutures were passed under the LADs without ligation. Animals were housed routinely following surgery. After ECG testing, rats that averaged QT-interval prolongation in three precordial leads were included into the model and QSYQ pharmacologic study. Then they were divided into 2 groups randomly, 8 in model group and 8 in QSYQ group. Meanwhile, 8 in sham-operated group were investigated together. The QSYQ group was treated for 28 days by daily irrigation stomach with total daily dosages of 2.33 g/kg of the concentrated QSYQ (Beijing university of Chinese Medicine, Beijing, China) dissolved in water as we did before [15, 16]. The sham-operated group and model group received the same volume water via irrigation stomach as the QSYQ vehicle. At the end of the study, all animals were anaesthetized using Isoflurane (Abraxis BioScience, Richmond Hill, ON, Canada) following an overnight fast. Heart tissue samples were excised parallel to coronary sulcus, 3 mm apart from cardiac apex. All samples were frozen in liquid nitrogen immediately for further examinations.

### 2.3. Preparation and Dose Consideration of Concentrated QSYQ

The QSYQ used in the present study was manufactured by Beijing University of Chinese Medicine (Beijing, China) using the six Chinese herbs at a composition of 460 g Radix Astragali Mongolici, 230 g salvia miltiorrhiza bunge, 160 g Flos Lonicerae, 160 g scrophularia, 140 g Radix Aconiti Lateralis Preparata, and 90 g Radix Glycyrrhizae as before [15, 16]. Briefly, following extraction with 95% ethanol, the residue of Radix Astragali Mongolici was mixed with all salvia miltiorrhiza bunge, Flos Lonicerae, *Scrophularia*, and Radix Glycyrrhizae, followed by extraction with hot water (twice, 2 hr each). The water extract was then concentrated to form a paste, and the ethanol was added for 24 hr, collected the filtration to form the final product. In present study, dosage of 2.33 g/kg was established as the same content at our previous study [15, 16].

### 2.4. Echocardiographic Assessment of Left Ventricular Function

Echocardiography was used to detect the left ventricular end-systolic diameter (LVEDs), left ventricular end-diastolic diameter (LVEDd), ejection fraction (EF), fractional shortening (FS), and other indicators. Vevo 2100 Imaging System (VisualSonics, Canada) with RMV 710B probe (21 MHz probe) was employed, which generates two-dimensional images at a frame rate of 300 to 500 frames/s. The LV dimension (LVD) was measured using m-model fractional shortening, and FS% was calculated using the following equation: FS% = [(LVEDd − LVEDs)/LVEDd] × 100.

### 2.5. Histology and Immunohistochemistry (IHC)

The ventricles were fixed in 4% paraformaldehyde, paraffin embedded hearts were sectioned at 200 mm intervals from base to apex, and serial sections of 4 mm were cut and placed on polylysine-coated glass slides. Tissue sections were deparaffinized and stained with Masson's trichrome reagent. An avidin-biotin-peroxidase complex commercial method (cell and tissue staining kit, R&D Systems, Inc., USA) was used for immunohistochemistry. Briefly, 4 mm thick paraffin wax sections were mounted on slides, which were dried for 30 minutes in an oven (60–70°C) and deparaffinized in xylene. The slides were then placed in changes of ethanol for 2 minutes each. Washing in buffer solution was performed between steps. The slides were then placed in 3% hydrogen peroxide for 15 minutes and then were subsequently incubated in avidin block for 15 minutes, biotin block for 15 minutes, primary antibody (p53, caspase-3, Phoenix Pharmaceuticals Inc., Germany; 1 : 200) for 12 hours at 4°C, and biotinylated secondary antibody for 1 hour. The reagent incubation was performed with streptavidin peroxidase for 15 minutes. A 1-minute Mayer's hematoxylin counterstain was used. The slides were dehydrated, cleared with xylene, and mounted with permanent mounting medium. Finally pictures were analyzed by IPP 6.0 software.

### 2.6. Determination of Plasma Superoxide Dismutase (SOD), Malondialdehyde (MDA), and NOX2 by Radioimmunoassay (RIA)

The plasma was homogenized in saline containing enzyme inhibitor (0.3 M EDTA-Na 10 ul, 0.34 M 8-hydroxyquinoline 10 ul, and 0.32 M dimercaptopropanol 5 ul) (1 ml blood) on ice. The homogenate was centrifuged at 8000 ×g for 10 min. The supernatant was used for determination of SOD, MDA, and NOX2 using an RIA kit (Beijing Kangyuan Ruide Biotechnology Co. Ltd., Beijing, China) following the instructions of the company.

### 2.7. Terminal Deoxynucleotidyl Transferase dUTP Nick End Labeling (TUNEL)

The cell apoptosis rate in the myocardium was determined by TUNEL according to the manufacturer's instructions (Roche Applied Science, South San Francisco, CA, USA). Six micrographs were randomly selected, and the numbers of healthy or apoptotic cardiomyocytes were counted. The TUNEL-positive percentage of apoptotic cells was considered as the percentage of the total numbers of cells [17].

### 2.8. Measurement of Indicators by Western Blot

Total protein from myocardium tissues was extracted and separated by sodium dodecyl sulfate-polyacrylamide gel electrophoresis (SDS-PAGE). Proteins were then transferred to a nitrocellulose (NC) filter membrane, blocked, and probed sequentially with primary antibodies against NOX4 and matrix metallopeptidase-2 (MMP-2). After incubation in the primary antibody, the membrane was incubated in an appropriate secondary antibody. After washing, the bound antibody complexes were detected using an electrochemiluminescence reagent, taking the GAPDH as internal reference.

### 2.9. Statistical Analysis

Statistical analysis was performed with the SPSS program package (SPSS version 17.0). All data were presented as mean ± standard deviation (SD). Statistical analysis was carried out on three or more groups using one-way analysis of variance (ANOVA) and Dunnett's test. The values of *P* < 0.05 were considered statistically significant.

## 3. Results

### 3.1. QSYQ Suppressed Heart Failure after MI

**Figure 1:**
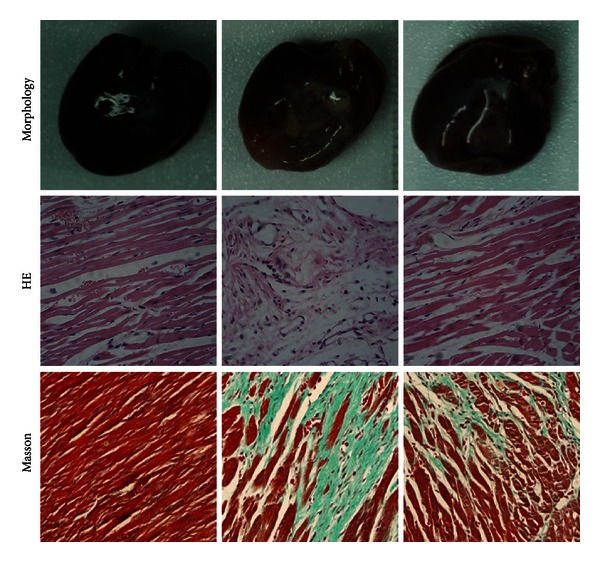
Morphology, HE, and Masson results in different groups.

In order to investigate the roles of QSYQ in protecting HF, we initially examined the heart morphological changes in different groups. 28 days after LAD ligation, hearts in model group rats presented with abnormal enlarged ventricular cavity (Figure 1). Pathological examination showed that the cardiac myocytes exhibited an irregular shape and arrangement with myocardial fibrosis. Masson trichrome staining showed fibrotic areas were significantly greater in model group than sham (Figure 1). Moreover, the number of cardiac myocytes was greatly reduced. Notably, QSYQ treatment significantly suppressed those events in the model (Figure 1) and lessened the fibrotic areas. 

### 3.2. QSYQ Upregulated Cardiac Function Related Parameters

**Figure 2:**
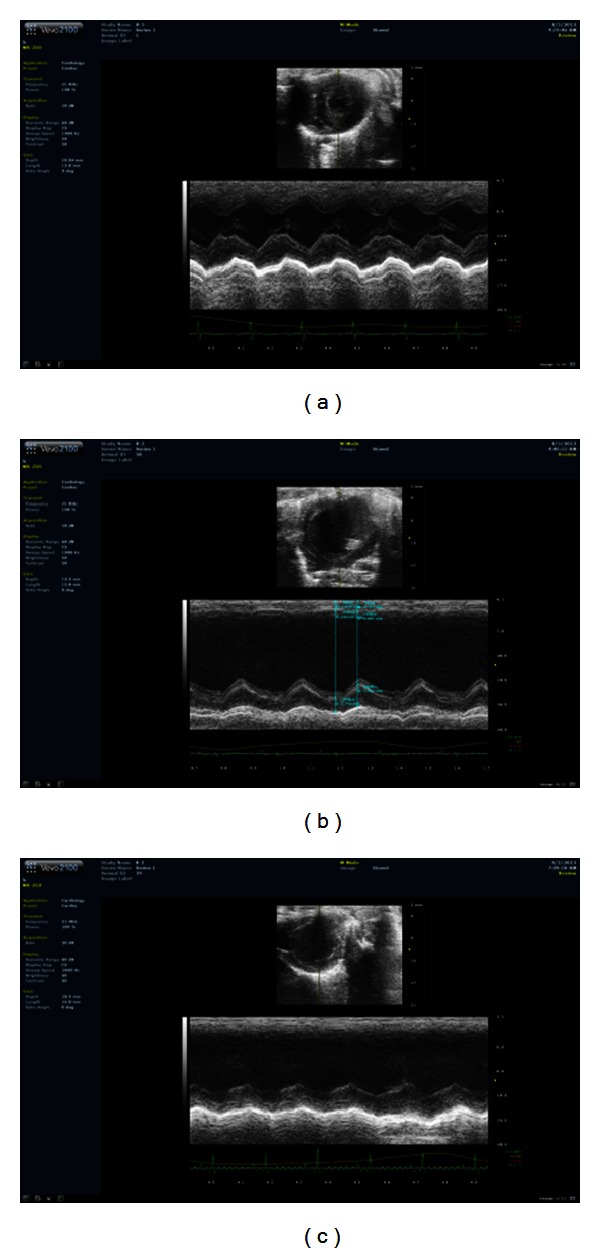
Cardiac function detected by echocardiography. (a) EF value, FS value, LVEDd, and LVEDs in sham-operated group. (b)  Increase in EF value, FS value and decrease in LVEDd, LVEDs in sham-operated rats. (c) Improvements in EF and FS in QSYQ group.

28 days after surgery, echocardiography showed that EF and FS values in the model group were significantly different (*P* < 0.05). EF value of model group rats dropped down to 50.97% compared with sham-operated group, accompanied by increase in LVEDd and LVEDs, suggesting a change of cardiac hypertrophy in this stage. After treated by QSYQ for 28 days, the EF value and FS value recovered by 33.88% and 46.50%, respectively; compared with model group, LVEDs was also less than model group, with a reduction of 17.65%, but LVEDd had no significant change with model rats. The ventricular wall in QSYQ group was still thicker than sham-operated group (Table 1, Figure 2).

### 3.3. QSYQ Treatment Inhibited Apoptosis of HF after MI

**Figure 3:**
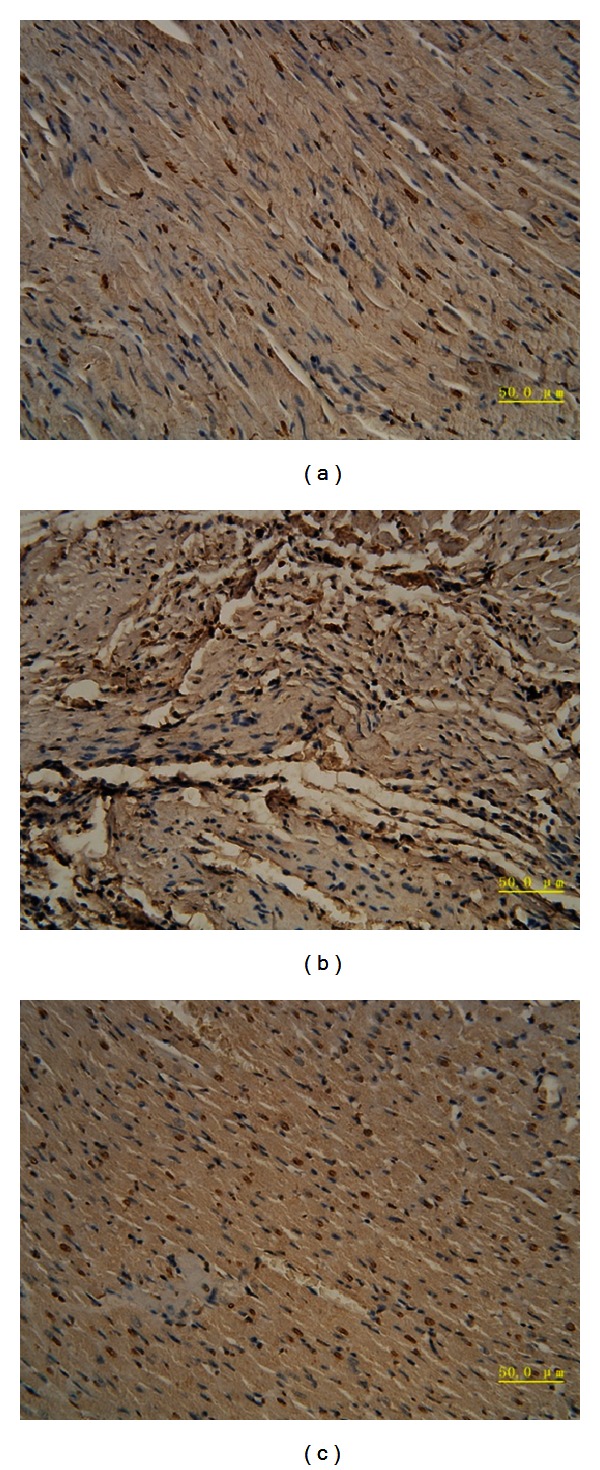
QSYQ inhibited cell apoptosis in the rat with HF. TUNEL analysis was carried out 28 days after the end of drug treatments. (a) The TUNEL-positive cells (apoptotic cells) in sham group. (b) Model; (c) QSYQ.

Cell apoptosis is one of the major outcomes of HF. Our previous study motivated us to further investigate the impacts of QSYQ on myocardial cell apoptosis. With the TUNEL assay, we found that increased numbers of apoptotic myocardial cells were presented in HF rats, whereas the QSYQ treatment dramatically decreased cell apoptosis rate (Figure 3).

**Figure 4:**
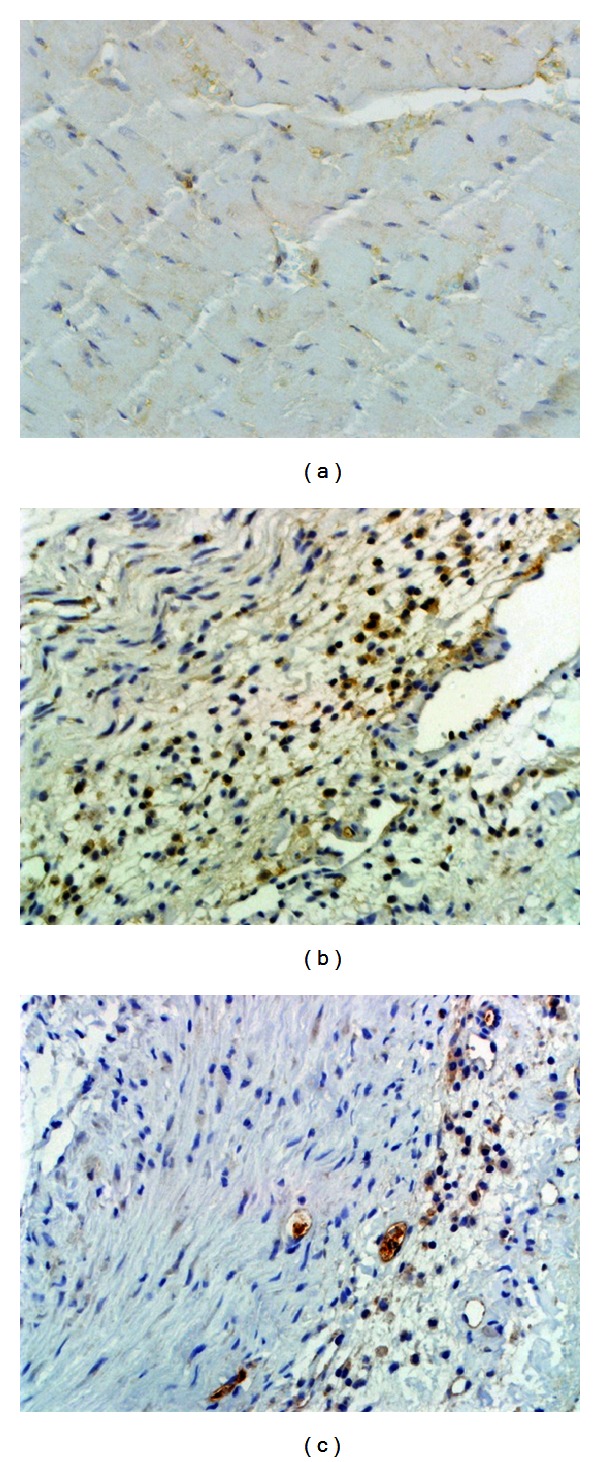
IHC results of p53 in different groups (×400): (a) sham; (b) model; (c) QSYQ.

**Figure 5:**
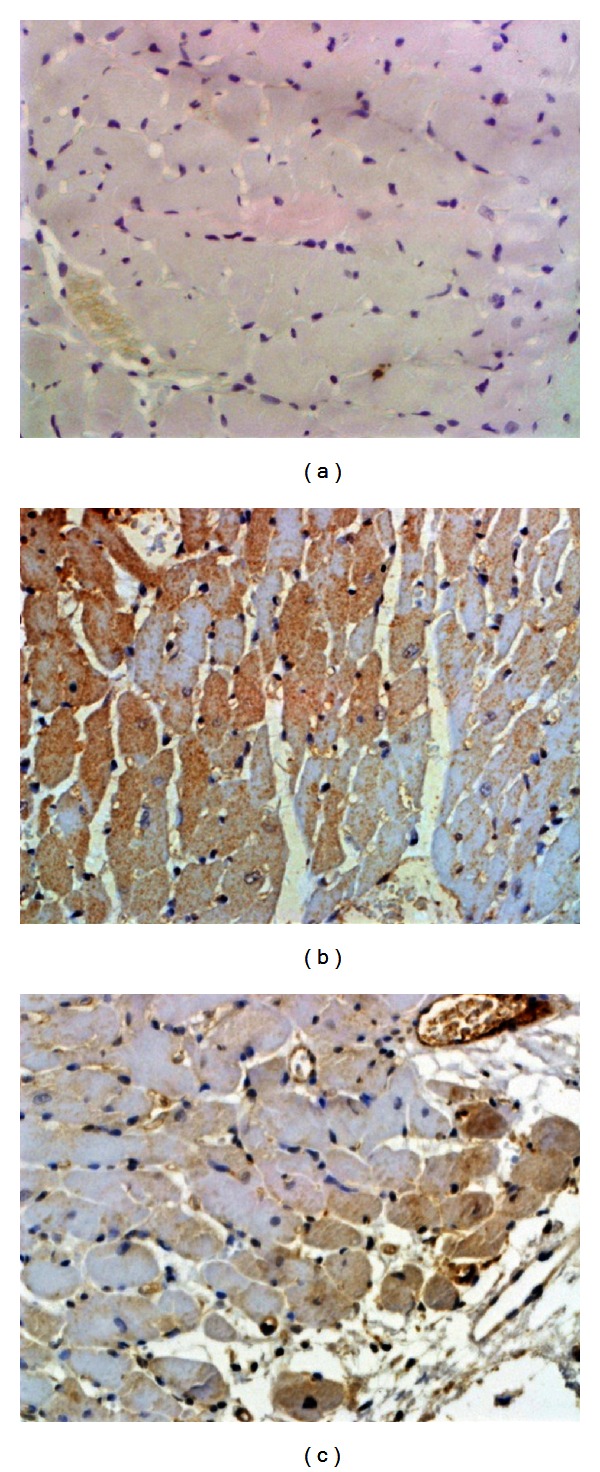
IHC results of caspase-3 in different groups (×400): (a) sham; (b) model; (c) QSYQ.

**Figure 6:**
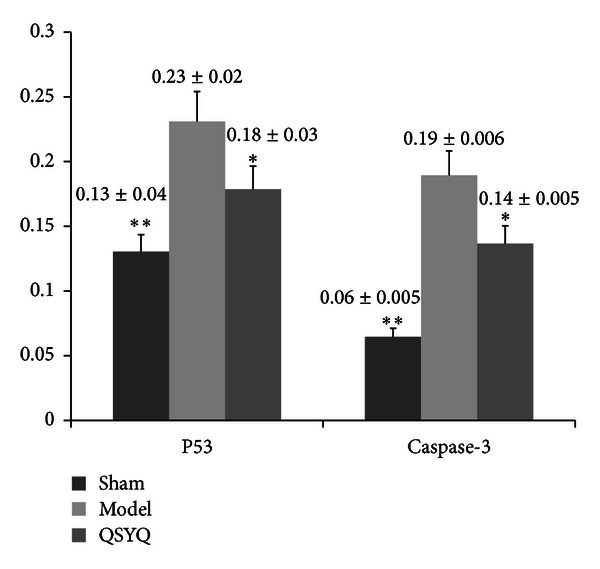
Quantification of apoptotic cell death. *P < 0.05 compared with model group, **P < 0.01 compared with model group.

To further confirm the results, p53 and caspase-3, which are the major indicators of apoptosis, were detected by IHC, respectively. As revealed in Figures 4, 5, and 6, compared with sham, p53 was significantly up-regulated as well as the caspase-3. Combined with TUNEL, all evidence indicates elevated degree of apoptotic changes in model group rats. QSYQ greatly suppressed cell apoptosis by p53 and caspase-3. Altogether, these results demonstrated that QSYQ can definitely inhibit cell apoptosis in HF. 

### 3.4. QSYQ Treatment Inhibited Oxidative Stress Levels in HF

The RIA of SOD showed that the plasma SOD in model group decreased by 32.88% (*P* < 0.05) compared with sham; the level of SOD in QSYQ group showed a 51.83% upregulation compared with model group (*P* < 0.05), which had no statistical significance when compared to the sham (Table 2). Results of MDA showed reverse changes. MDA in model group increased by 33.62% (*P* < 0.05) compared with sham, while the level of MDA after QSYQ treatments showed a 31.68% downregulation compared with model group (*P* < 0.05), which is almost same to the level of sham (Table 2). NOX2 showed a similar outcome as MDA. Its level in model group is up-regulated by 29.79%, and a 24.45% reduction was detected in QSYQ group compared with model group. 

**Figure 7:**
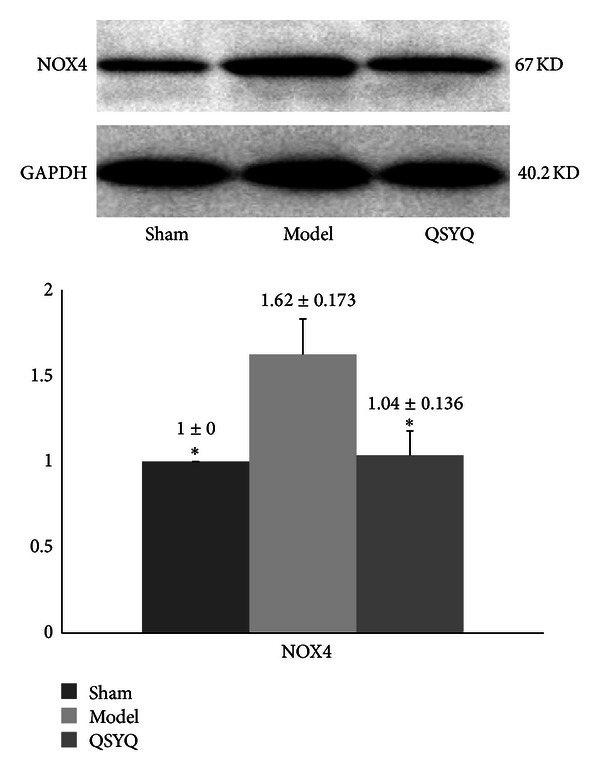
The Western blot results of NADPH oxidase (NOX4) in different groups. QSYQ: QiShenYiQi.

The Western blot showed that; the NOX4 in model group increased by 62.00% (*P* < 0.05) compared with sham, the level of NOX4 in QSYQ group showed a 35.80% reduction compared with model group (*P* < 0.05) (Figure 7). 

### 3.5. QSYQ Treatment Inhibited the Level of MMP-2

**Figure 8:**
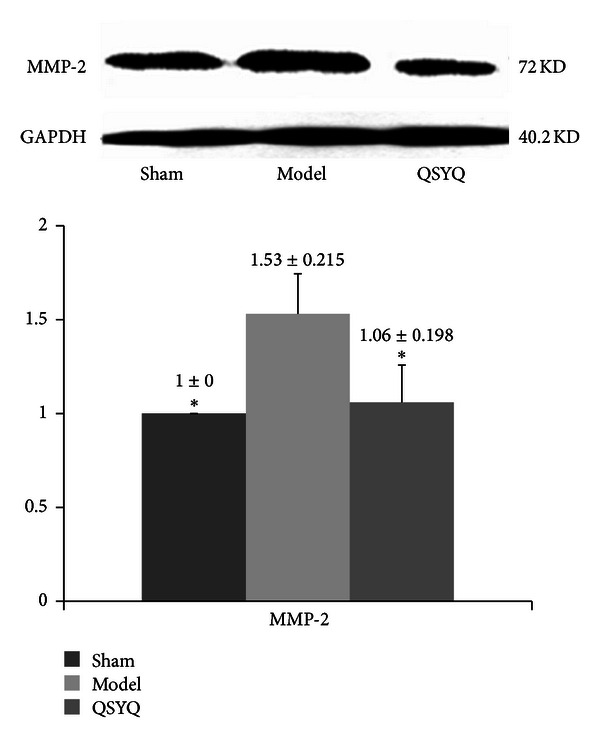
The Western blot results of MMP-2 in different groups. QSYQ: QiShenYiQi.

MMP-2, as a critical prognosis for heart failure [18], was also detected in present study. The Western blot of the cardiac MMP-2 in model group increased (1.53 ± 0.215, *P* < 0.01) compared with sham-operated group (1.00 ± 0.000), while, treated by QSYQ for 28 days, the level of MMP-2 showed a reduction compared with model group (1.06 ± 0.198, *P* < 0.05), which had no statistical significance compared to the sham (Figure 8). 

## 4. Discussion 

A variety of stimuli, such as oxidative stress [19] and inflammatory factors [20], have been suggested to trigger apoptosis and eventually caused myocardial modeling during HF. First, the stressed endoplasmic reticulum (ER) induces the activation of oxidative stress including ROS and NADPH [21] and subsequently leads to cardiac myocytes apoptosis [22]. There is evidence indicating that the expressions of NOX2 and NOX4 are markedly changed, accompanied by the activation of apoptosis [23]. Consistent with previous studies, here we found changed expressions of SOD and MDA in HF model, suggesting that induction of oxidative stress occurred during HF after MI [24]. Moreover, the two isoforms of NADPH oxidases were believed to cause apoptosis by inducing ROS [25]. NOX2 and NOX4 are major sources of ROS in endothelial cells and are implicated both in vasodilator dysfunction and in the modulation of redox-sensitive signaling pathways [26], and the activation of NOX2 and NOX4 showed distinct functions. NOX2 is normally quiescent and is acutely activated by stimuli such as G-protein-coupled receptor (GPCR) agonists (e.g., angiotensin II, endothelin-1) and cytokines to initiate enzyme activity [27]. Some studies indicated that NOX2 activation contributes to angiotensin-II-induced cardiomyocyte hypertrophy. NOX2 contributes to myocyte death under stress situations and plays important roles in postmyocardial infarction remodeling, in part by modulating matrix metalloprotease activity. In contrast to NOX2, NOX4 is constitutively active at a low level and induces other effects in the heart under chronic stress, for example, by inducing the cell death signaling [28]. Therefore, NOX4 was regarded as an inducible isoform. That is to say, NOX2 plays an important role in mediating angiotensin-II-induced cardiac hypertrophy through MMPs, while NOX4 mediates cardiac hypertrophy and heart failure in response to pressure overload mainly by apoptosis [29]. 

**Figure 9:**
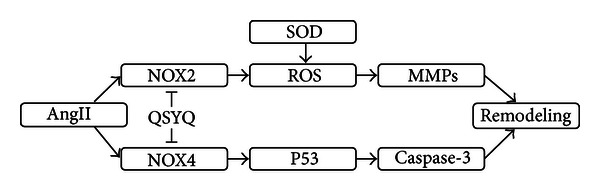
Potential mechanism of QSYQ attenuates oxidative stress and cell death signaling pathways in heart failure rats.

Interestingly, in present study, the QSYQ can significantly downregulate the level of both oxidative stress and apoptosis, indicating a synergetic efficacy on HF. Moreover, NOX2 and NOX4 showed significant increase in model group. Both echocardiographic and MMP-2 results all indicated a cardiac hypertrophy after being operated for 28 days, while QSYQ can improve cardiac remodeling through counter-acting these events. Combined with our previous study and the references [16, 27], AngII-NOX2-ROS-MMPs pathway seems to be the critical target for QSYQ to reduce the remodeling. Furthermore, NOX4 is another potential targets to inhibit the p53 and caspase-3 [26], thus to reduce the apoptosis and hypertrophy and eventually provide a synergetic cardiac protective effect (Figure 9).

To sum up, this paper presents a multitarget pharmacological study of Chinese herbal formula. The TCM with multiple chemical components targets on multiple proteins [30], which may produce greater synergetic efficacy and fewer side effects [31]. And the results also show “therapeutic” QSYQ administration which can reduce the oxidative stress and apoptosis in NOX2 and NOX4 pathway, respectively, further proved the beneficial effects of QSYQ. In conclusion, administration of QSYQ could attenuate LAD-induced HF, oxidative stress, and apoptosis partly through the NOX2 and NOX4 pathways, which can treat CHD efficiently and safely. 

But some problems still exist. For example, how the NOX4 affected the p53 pathway was not investigated. Moreover, whether the NOXs shared same pathway, or the interactive effects between them were not taken into consideration. Improvements should be made in our future work.

## Figures and Tables

**Table 1:** Echocardiography of rats in each group.

Group	*N*	LVEDd (CM)	LVEDs (CM)	FS (%)	EF (%)
Sham	8	0.740 ± 0.047**	0.350 ± 0.055**	52.470 ± 5.423**	87.41 ± 4.271**
Model	8	1.050 ± 0.107^▲▲^	0.850 ± 0.078^▲▲^	18.710 ± 1.675^▲▲^	42.86 ± 3.110^▲▲^
QSYQ	8	0.950 ± 0.128^▲▲^	0.700 ± 0.203^▲▲∗^	28.071 ± 11.879^▲▲∗^	57.38 ± 18.519^▲▲∗^

^▲▲^ *P* < 0.01, versus sham-operated group; *P < 0.05, **P < 0.01, versus model group.

**Table 2:** Levels of oxidative stress indicators in different groups.

Group	*N*	SOD (U/mgprot)	MDA (nmol/mgprot)	NOX2 (ng/mL)
Sham	8	145.870 ± 14.807**	5.740 ± 0.992*	5.640 ± 0.701*
Model	8	97.940 ± 3.388^▲▲^	7.670 ± 0.896^▲^	7.320 ± 1.341^▲^
QSYQ	8	148.700 ± 32.041**	5.240 ± 1.736*	5.530 ± 0.335*

Compared with model, *P < 0.05, **P < 0.01; compared with control, ^▲^ *P* < 0.05, ^▲▲^ *P* < 0.01.
